# A Text Book of Diseases of Women

**Published:** 1897-11

**Authors:** 


A TEXT BOOK OF DISEASES OF WOMEN—By Charles B. Penrose, M. D., Ph. D., Professor of Gynecology in the University of Pennsylvania; Surgeon to the Gynecean Hospital, Philadelphia, 529 pages, with numerous illustrations. Price, cloth $3.50 net. W. B. Saunders Publisher, 925 Walnut Street Philadelphia, 1897.

This book is designed especially for medical students. The author has, as a rule, omitted all facts of anatomy, physiology, and pathology which may be found in the general text books upon these subjects. He has in most instances recommended one plan of treatment for each disease, in this way avoiding the confusion of the student who consults this book for practical guidance. This especial feature of this book commends it to the student and physician, and will be duly appreciated by them. There is nothing which so confuses the medical student as a long list of remedial agents or procedures that may be tried, in a given disease, thus suggesting the probable failure of one or all of them. Many medical men of today doubtless remember a certain leading text-book on the theory and practice of medicine, in use during their student days, in which the author quoted the recommendations of doctors A. B. C. and so on, but never told the student how to treat any disease with the expectation of effecting a cure. Such a book is enough to discourage the student, and make of him an unbeliever in the science of medicine. This volume will inspire confidence by its positive way of presenting treatment; and its conciseness, together with the fact that it contains the latest and best teachings of gynecology, will make it a favorite with medical students and practitioners.

